# Reflections on youth partnership in a randomized controlled trial of an intervention to optimize transition from pediatric to adult care in inflammatory bowel disease

**DOI:** 10.1016/j.hctj.2025.100111

**Published:** 2025-07-05

**Authors:** Brooke Allemang, Ashleigh Miatello, Pranshu Maini, Joshua Eszczuk, Claudia Tersigni, Samantha Micsinszki, Sneha Dave, Natasha Bollegala, Nancy Fu, Kate Lee, Samantha J. Anthony, Melanie Barwick, Eric I. Benchimol

**Affiliations:** a Child Health Evaluative Sciences, SickKids Research Institute, The Hospital for Sick Children, Toronto, Canada; b Office of Patient, Family, and Community Engagement, The Hospital for Sick Children, Toronto, Canada; c Factor-Inwentash Faculty of Social Work, University of Toronto, Toronto, Canada; d Faculty of Kinesiology, University of Alberta, Edmonton, Canada; e Schulich School of Medicine and Dentistry, Western University, London, Canada; f School of Rehabilitation Science, McMaster University, Hamilton, Canada; g CanChild Centre for Childhood Disability Research, McMaster University, Hamilton, Canada; h Generation Patient, United States; i Division of Gastroenterology, Department of Medicine, Women’s College Hospital, University of Toronto, Toronto, Canada; j Division of Gastroenterology, Department of Medicine, University of British Columbia, Vancouver, Canada; k Crohn’s and Colitis Canada, Toronto, Canada; l Department of Psychiatry, University of Toronto, Toronto, Canada; m Dalla Lana School of Public Health, University of Toronto, Toronto, Canada; n Division of Gastroenterology, Hepatology and Nutrition, SickKids Inflammatory Bowel Disease Centre, The Hospital for Sick Children, Toronto, Canada; o Department of Paediatrics and Institute of Health Policy, Management and Evaluation, University of Toronto, Toronto, Canada; p ICES, Toronto, Canada

**Keywords:** Adolescents, Inflammatory bowel disease, Patient engagement, Patient-oriented research, Transition to adult care, Young adults

## Abstract

**Background:**

Patient engagement allows for developing health interventions and research studies that align with the needs of people living with chronic diseases. Actively involving people with lived experience as partners in the design and governance of interventional trials is gaining prominence. However, there is a dearth of literature exploring the processes and procedures of patient partnership in various stages of randomized controlled trials (RCTs) with adolescents and young adults (AYAs) with inflammatory bowel disease (IBD), specifically.

**Methods:**

This reflective viewpoint outlines how AYA patient partners were involved in a RCT of an intervention to optimize transition from pediatric to adult care in IBD through a Youth Advisory Panel. To achieve this objective, we: i) gathered information from eight members of the study team via virtual discussions, ii) reflected on our experiences collaborating with patient partners in the RCT, and iii) reviewed relevant documents, including meeting minutes. Discussion transcripts, reflections, and documents were reviewed by two academic researchers and one AYA patient partner to identify the varying levels of involvement among AYA patient partners in the trial. The engagement strategies implemented to maximize the translational impact of the multimodal intervention and inform patient-centered care across various stages of the RCT were also examined.

**Results:**

The processes, outputs and impacts of AYAs’ contributions in priority setting and planning, intervention design and refinement, study execution, dissemination, and post-trial intervention implementation and dissemination were identified. AYA patient partner motivations for involvement in the RCT and key learnings from this partnership were uncovered.

**Conclusion:**

AYA patient partners’ contributions to the RCT were impactful, from study conceptualization to dissemination. Their involvement ensured the intervention's relevance, usability, and youth-friendliness, informed data analysis, and enhanced knowledge translation through co-creation. Future work in this field could involve evaluating the impact of patient engagement on AYA patient partners, research teams, and research outcomes in RCTs.

## 1 Introduction

Patient engagement in research refers to individuals with lived experience meaningfully contributing to research design, conduct, and dissemination.^1^ Evidence suggests that involving patients across the research spectrum results in more relevant and impactful studies that align with the priorities of the communities affected by the phenomena under investigation.^2^, ^3^ The roles of patients and researchers vary depending on the resources available for engagement, project timelines, and partnership goals. Often, partnership processes are guided by frameworks which outline different engagement approaches, opportunities for impact, and core values to clarify expectations for both patients and researchers.^4^, ^5^, ^6^

Patient engagement frameworks offer research teams valuable tools, foundational principles, and guidance for involving people with lived experience in research and avoiding common pitfalls such as tokenistic approaches and a lack of clarity about scope of involvement.^7^, ^8^ One such framework, the International Association of Public Participation (IAP2) Spectrum of Public Participation, identifies levels of partnership between people with lived experience and researchers that exist along a continuum, from patients being informed, consulted, involved, collaborating, and empowered.^5^ A table outlining IAP2’s levels of partnership and the extent of patient involvement according to level is provided in Supplementary Table 1. Hart’s Ladder of Youth Participation is a youth-specific framework outlining youths’ quality of participation in programs or research initiatives.^9^ It explicates how decision-making power is shared amongst youth and adults, with tokenism and limited capacity for youth impact at the lower rungs, to youth-initiated decisions which are supported by adults at the upper rungs of the ladder.^9^ Clarifying the scope of patient involvement in research studies, the engagement strategies employed, and the resulting impact on research processes is important for providing transparency to patient partners and reporting to the public upon study completion. While patient engagement in chronic disease research is gaining prominence,^10^ further details about engaging patients in randomized controlled trials (RCTs) are required to understand the impact of patient involvement and promote replicability of engagement processes within these types of studies.

Inflammatory bowel diseases (IBD) comprise chronic gastrointestinal disorders with two main sub-types of Crohn’s disease and ulcerative colitis. The symptoms of IBD significantly impact patients’ health, psychosocial functioning, and overall quality of life.^11^, ^12^ People living with IBD can play important roles in contributing to the design and conduct of research studies in this field, given their understanding of the daily realities of managing IBD and patients’ needs.^3^, ^10^ Literature examining patient engagement in IBD research and its impact on study outputs has increased in recent years.^3^, ^10^, ^13^, ^14^, ^15^, ^16^ Most studies to date have adopted consultation methods to bring patients’ perspectives to developing research tools, including questionnaires or guidebooks.^10^ Of note, only two studies described in a recent scoping review were RCTs.^10^ This suggests that research and commentaries are needed to clarify engagement processes, experiences, and impacts in IBD RCTs, including intervention design and refinement, participant enrollment and engagement, and knowledge translation. Further, methods for involving adolescent and young adult (AYA) patient partners with IBD in designing, conducting, and disseminating healthcare transition-related research are lacking.

The transition from pediatric to adult healthcare for AYAs with IBD can be complex given the shift from a family-centered to a patient-centered environment, expectations for greater autonomy over disease self-management, co-occurring life transitions, and the prominence of psychosocial stressors during this period.^17^, ^18^, ^19^, ^20^ The experiences and perspectives of AYAs with IBD can contribute to developing healthcare interventions and research studies targeting the barriers associated with transition. A recent patient-driven analysis of the complexities of transition for AYAs with IBD highlights the inherent value of their expertise and voices in determining future research and clinical priorities for this population.^21^ Given the importance of involving AYAs with IBD in designing and evaluating transition interventions to address transition-related challenges, our team engaged with AYA patient partners in conducting a multi-site RCT called the IBD-Transition Adolescent and Young Adults implementatioN (IBD-TrAYN) Trial.^22^ In this reflective viewpoint, we describe and collectively reflect on the roles AYA patient partners adopted during the RCT, the processes used to engage them, and the impact of these efforts on the study and AYA patient partners themselves.

## 2 Overview of the IBD-TrAYN Trial in which patients were engaged as partners

AYA patient partners were engaged in the development, design, and conduct of an ongoing, multi-site RCT examining the impact of IBD-TrAYN which is a multimodal transition intervention on quality of life, psychosocial, and functional outcomes for youth with IBD at three Canadian tertiary care centers (ClinicalTrials.gov NCT05221281).^22^ Study participants, who were distinct from AYA patient partners, were randomized to the study’s intervention or control arm (standard of care for transition). The intervention was comprised of four core components: i) support from a transition navigator (a nurse or social worker) whose role was to identify psychosocial and educational gaps with, ii) individualized assessments to establish transition-specific goals, engage in relevant skill-building activities, and act as a point of contact during transition for two years,^22^ iii) self-paced online skill-building modules, and iv) an online education program with topics focused on IBD management, resilience, and healthcare transition, for the duration of the study. ^17,22^ Ethical approval for the IBD-TrAYN Trial was granted by the Research Ethics Boards of the Hospital for Sick Children, Children's Hospital of Eastern Ontario, and BC Children's Hospital.

## 3 Patient engagement in the *[Intervention Name]* trial

### 3.1 Youth advisory panel

AYA patient partners living with IBD were involved in the IBD-TrAYN Trial from project conception to dissemination through a Youth Advisory Panel (YAP).

#### 3.1.1 Purpose of the youth advisory panel

The YAP was developed to enhance the effectiveness and uptake of the study intervention and to ensure a comprehensive understanding of patient-defined priorities surrounding healthcare transitions.

#### 3.1.2 Youth advisory panel composition

The YAP comprised 12 youth with IBD aged 15–24 from across Canada who were not RCT participants. YAP members were recruited via email through the Young Adult Community for Crohn’s and Colitis, a patient-founded initiative for young adults with IBD chaired by patients, and Crohn's and Colitis Canada, a national community-based organization supporting individuals with IBD, which was also a partner and funder of the trial.

#### 3.1.3 Youth advisory panel engagement structure

**Fig. 1:**
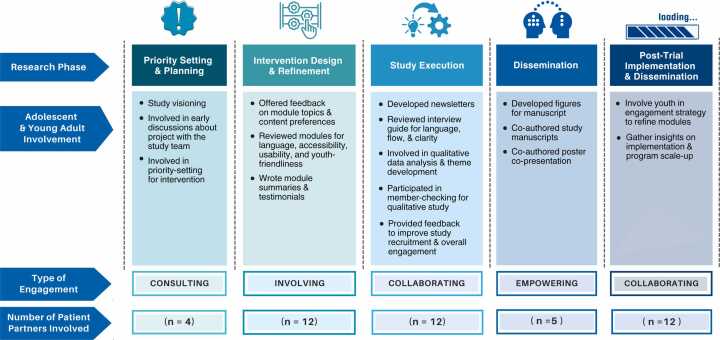
Adolescent and Young Adult Involvement According to Research Phase. Note: Types of engagement are adapted from the IAP2 Spectrum of Public Participation^5^.

The YAP’s inaugural virtual meeting was held in December 2021, and the group subsequently convened bi-annually and on an ad-hoc basis, with meetings lasting one hour. Between 2021 and 2024, the YAP have met virtually six times and will continue to meet until the completion of the trial in 2026. The YAP was facilitated with the support of a Chair member (CT initially and then SM) living with IBD. The role of the YAP Chair was to bridge the YAP members to the trial’s principal investigators and thus, the YAP Chair led the bi-annual YAP meetings and attended the monthly principal investigator meetings to share members’ comments and feedback. Representatives from Crohn's and Colitis Canada were instrumental to the organization of YAP meetings, as they took meeting minutes and organized meeting dates/times. YAP members were volunteers who were not offered financial compensation for panel participation. However, they received electronic gift cards (facilitated by Crohn's and Colitis Canada) that could be used for snacks during some meetings, reference letters from the study team (as needed), an acknowledgement in study publications, co-presentation and/or co-authorship opportunities, and training/mentorship in research methods, including qualitative analysis. The YAP were consulted at group meetings and between meetings asynchronously via document reviews or requests for written feedback. Fig. 1 elucidates the many ways the AYA patient partners, who were members of YAP, were involved in the RCT. The engagement strategies employed varied based on the study phase and AYA preferences. Below, we describe AYAs’ roles and levels of engagement across different phases of the research process and the subsequent or concurrent impact of their involvement in the study (See Fig. 1).

### 3.2 Methods for gathering team reflections about patient partner engagement

To understand how patient partners were involved in the IBD-TrAYN Trial, we: i) gathered information from eight members of the study team, including one academic researcher, six AYA patient partners, and one representative from the community organization who funded the RCT, ii) reflected on our experiences collaborating in the RCT, and iii) reviewed relevant documents. Five virtual discussions with the eight individuals noted above were facilitated by the first two authors (BA*, AM*) on Microsoft Teams and transcribed verbatim for notetaking purposes. In these discussions, team members were invited to reflect on the methods and processes for involving patient partners in the RCT, the impact(s) of involvement, and their learnings about patient engagement. One set of questions was used for the researcher and community organization representative discussions, and another set of questions was used for all discussions with the AYA patient partners, which focused on their perceptions of engagement, motivations to get involved in research, and how the engagement opportunity impacted them. Sample questions for the researcher/community organization representatives included, “From your perspective, how were AYA patient partners involved in developing the RCT intervention?”, and “From your perspective, how did involving AYA patient partners in the RCT impact the study as a whole?”. Questions for the AYA patient partners included, “What phases of the RCT were you engaged in as an AYA patient partner?”, “How were you engaged as an AYA patient partner throughout the trial?”, and “What impact did these activities have on your personal growth and learning (if any) or on your contribution to a larger patient care impact?”. Transcripts and notes from these meetings were reviewed by two academic researchers (BA, AM) and one AYA patient partner (PM) to identify types of patient involvement across study phase and key findings about the process of engagement in the RCT through a collective review of the information obtained. The three team members involved in consolidating the findings engaged in independent reviews of the transcripts, notes, and meeting minutes, held collective discussions about the prominent concepts, and came to consensus about the patient partner roles and impacts of engagement. Each team member’s unique perspective was emphasized throughout this process, as is common in collaborative projects which involve academics and individuals with lived experience to benefit from differing views.^23^ Select verbatim quotes from patient partners shared in these discussions are presented throughout the article to provide supporting evidence about key processes, impacts of engagement, and learnings in their own language. In addition, meeting minutes from the initial IBD-TrAYN Trial planning meeting and ongoing YAP meetings were scanned to ensure accuracy in reporting the activities of YAP members. Finally, two YAP members (PM, JE) contributed their perspectives about YAP members’ motivations to become involved as patient partners in writing gleaned through YAP meetings and their own reflections.

### 3.3 Patient partner engagement in priority setting & planning

During the planning stage of the trial, three AYA patients with IBD and one parent were invited to a day-long planning session. These individuals were suggested by physician members of the planning committee given their lived experiences and commitment to advocacy work in the IBD field. This meeting included clinicians, scientists, advocacy partners, youth and family member representatives. During this session, the group discussed experiences and priorities for optimal transition care and core components that should be included in the intervention. The AYA patient and parent partners were invited to share their experiences transitioning from pediatric to adult IBD care, highlighting the challenges they faced, the gaps they encountered, and areas where improvements could be made. Afterwards, there was an extensive question and answer period to gather more detailed insights that would later inform the intervention components. For example, the AYA patient and parent partners described the importance of developmentally relevant education modules and access to a support person who was not their physician (e.g., transition navigator role). They emphasized the importance of supporting AYAs’ psychosocial well-being. All proposed improvements for transition became core components of the developed intervention.

Once the trial was funded, a research coordinator (RC) was hired at the lead study site. This individual was one of the AYA patient partners who had participated in the study planning session and was a founder and co-chair of a national Young Adult Community for Crohn’s and Colitis. This individual initially joined the study as an RC but later transitioned to a managerial role, overseeing the implementation of the study at two additional sites. This progression underscores the consistency maintained across study sites and highlights how the patient engagement component was further integrated into other sites, given this individual’s direct involvement in their operation. The RC recruited other AYAs from the Young Adult Community for Crohn’s and Colitis to join a study-specific YAP. The YAP developed a charter to set expectations, goals, and responsibilities and to outline communication preferences. One of the first tasks for the YAP was to offer insights about the relevant topics and delivery modes for the skills-building and e-learning modules.

### 3.4 Patient partner engagement in intervention design & refinement

Once the multimodal transition intervention for the RCT had been drafted, the YAP provided invaluable feedback on its content and delivery mode using synchronous and asynchronous methods, enhancing its youth-friendliness and relevance to study participants (See Fig. 1). YAP members were provided access to 21 education e-modules and six mandatory and eight optional skill-building e-modules in test mode before their trial launch. They were invited to review the modules and offer input on the overall usability. YAP members were given two options for providing feedback to allow them autonomy over how they chose to communicate their input. First, they received a template by email that asked specific, prompting questions about each module’s title, language, content, engagement, and accessibility, allowing them to share their thoughts in written form and at their own pace. Second, a virtual YAP meeting was held, where members were invited to share their thoughts on the e-modules and videos verbally or using the chat box in real-time, stimulating a fulsome discussion. Most members used the template to document their thoughts comprehensively, and the study team summarized the collective feedback received to implement feasible changes to the e-modules.

Based on the YAP’s input, several additions were made to the curriculum, including knowledge checks to better engagement, formatting changes for improved accessibility (e.g., bolding/highlighting key terms, increasing font size, adding closed captions to videos), dropdown menus to assist in navigation, and additional content relevant to participants (e.g., tips for finding a family doctor, tax credits for ostomies). YAP members also suggested that each module begin with a content summary and an estimated completion time to assist with participant engagement. Subsequently, YAP members drafted short module summaries in lay language that were included in the platform. During a reflective discussion with the YAP, members expressed their appreciation for being involved as key decision-makers with “an equal seat at the table” and felt their feedback was taken seriously by the research team to help enhance the intervention's usability.

### 3.5 Patient partner engagement in study execution

During study execution, the RC reviewed all patient-facing documents for accessibility, and YAP members partnered with the study team to promote participant uptake in the RCT and to develop a qualitative sub-study. Regarding study initiation, the RC edited all study documents and consent forms to ensure that they were appropriate when patient-facing and relayed feedback received during discussions with patients and their families back to the study team. The RC then implemented these changes at the lead study site and subsequently made changes prior to ethics board submission at other sites. Once the study was launched, the YAP collaborated in determining how to increase participant retention. Given that the skills-building e-modules and education curriculum were offered digitally, the YAP was involved in brainstorming strategies to support participant engagement in the self-paced components of the intervention. During YAP meetings, members suggested sending reminder emails to participants highlighting the value of the e-modules. YAP members drafted brief testimonials about each module’s key message and impact on their knowledge from their perspectives. These first-hand accounts were intended to help convey the benefits of the e-modules and encourage participant engagement. The reminder emails highlighted two modules, including the testimonials, and were distributed monthly to the intervention arm participants in the RCT.

The objectives and interview guide of a qualitative sub-study embedded within the RCT (the results of which are published elsewhere^17^, ^24^) were shared with the YAP for consultation during a virtual meeting. YAP members endorsed the importance of gathering RCT participants’ perspectives on the intervention, how IBD affects their mental health, and their transition experiences. They offered verbal input on the interview guide's language, flow, and youth-friendliness. Their suggested additions, including more probes, helped improve the guide and made the questions clearer and easier for participants to understand. For instance, one YAP member recommended adding more specificity to the question “What do you need from healthcare providers to help you prepare for transition?” by including probes, like “What do you need in a clinic visit?”, “What do you need when learning to plan your day-to-day care for your health at home?”, and “What do you need when learning how to deal with healthcare administrative tasks that you must do as a patient (dealing with insurance, managing your prescriptions, booking your appts)?”. These probes were used to glean more information from participants about their daily transition experiences and needs, contributing to richer first-person accounts.

### 3.6 Patient partner engagement in dissemination

The YAP collaborated in developing dissemination outputs as co-authors on two qualitative manuscripts^17^, ^24^ and two poster presentations during the RCT. Once a subset of interviews had been conducted with trial participants, a consultation was held with the YAP to report on the progress of the qualitative sub-study and the impact of their input on the interview process. Additionally, preliminary concepts emerging from the codes two study team members had identified were presented to the group to learn how these resonated with the YAP. Members found the themes related to mental health during transition to adult care particularly poignant, and many felt the illustrative quotes presented reflected their experiences. This formed the basis for the first of two qualitative manuscripts arising from the dataset, specifically focused on the mental health experiences of youth with IBD during transition to adult care.^17^ Through a collaborative discussion, the YAP was involved in an informal member-checking exercise, where they offered input on interpreting the qualitative data. One YAP member provided extensive feedback on preliminary themes and was empowered to re-design two figures for the manuscript to enhance their impact and visual appeal. This YAP member assumed a co-author role in the first manuscript and was a co-presenter on a conference poster to reflect their contributions to the project. Later, a third consultation was held with the YAP, given the richness of the qualitative data and the desire to share findings regarding participants’ perceptions of the multimodal transition intervention. Given the YAP’s engagement throughout the qualitative consultations, the qualitative study lead invited YAP members to partner in developing the second qualitative manuscript as co-authors.^24^

Five of the 12 YAP members expressed interest in actively engaging as co-authors in developing the second qualitative manuscript. They joined a smaller working group, the YAP Qualitative Working Group, to bring their perspectives to data analysis, interpretation, and knowledge translation. The qualitative study lead (BA) met individually with each interested YAP member to understand their hopes for engagement, motivations for becoming involved, preferred methods of providing feedback (e.g., written vs. verbal), desired meeting structure, accessibility needs, and non-financial compensation considerations (e.g., mentorship, reference letters, networking). Once oriented to the opportunity, this group convened bi-weekly through virtual 1.5-hour meetings over two months (March to May 2024). Meetings were scheduled at times that suited YAP members’ schedules and were recorded for those who could not attend. Meeting agendas, materials for review, template feedback forms, and guiding discussion questions were emailed to YAP Qualitative Working Group members one week before each meeting and were housed on a shared drive accessible to all members. Providing resources such as agendas, materials for review, and discussion prompts helped alleviate the pressure on attendees to respond in meetings. This allowed them to think more freely and thoughtfully in advance, leading to organically developed ideas before the meeting rather than in the moment. AYAs also completed a “methods of contribution tool” developed for this project to articulate the areas of the manuscript they hoped to be involved in and the types of roles they wanted to assume. This provided them with autonomy over their level and scope of involvement. Each meeting began with icebreaker questions to support rapport building and “neutralize power imbalances," which were found to “improve group dynamics” (YAP member).

The qualitative study lead provided members with a study overview and data analysis progress, was responsible for coding the qualitative interviews, and presented preliminary codes to the YAP Qualitative Working Group for discussion. During the second group meeting, the qualitative study lead delivered a training session on coding, thematic analysis, and using NVivo software for qualitative research. This session was well-received by the YAP, as it “helped [members] develop a foundational basis of what exactly they were contributing to” (YAP member) and offered a skill set potentially useful in future endeavors. Through facilitated group conversations, members were involved in theme development by sharing their perspectives about grouping similar concepts and identifying salient codes and illustrative quotes to include in the manuscript after reviewing the de-identified data. They then collaborated in interpreting the data by considering how the emergent themes mapped onto their experiences and the existing literature and offered suggestions on grouping sub-themes. Next, members self-selected manuscript preparation tasks of interest and roles were determined based on their strengths. Each YAP member engaged in writing or revising sections of the manuscript. Insights gleaned through reflective discussions with the YAP expanded the qualitative study lead’s understanding of the data. For instance, relationships, community, and peer support during transition were raised as important by YAP members within the data and their life journeys. A sub-theme focused on relationality was subsequently developed and co-written by a YAP member. Another YAP member, skilled in graphic design, independently created two figures displayed in the manuscript's results section, exemplifying creativity, leadership, and empowerment. Through a reciprocal learning process, this YAP member informed the qualitative study lead and other YAP members about the power of visuals in communicating complex ideas and the process of developing them using Canva.

Engaging the YAP and YAP Qualitative Working Group in dissemination planning and execution resulted in clear and visually appealing figures, results that resonated with people with lived experience, insights regarding study limitations, and a more nuanced interpretation of findings than possible with the research team alone. Their perspectives offered a more holistic understanding of the interview transcripts/participant narratives that researchers without lived experience might have missed, thereby supporting more grounded coding and theme development that reflected real-world patient priorities. Active collaboration with the YAP subsequently enhanced the relevance of the intervention to people living with IBD, and the research findings to the broader community.

YAP members also expressed the impact of their involvement on their personal growth, knowledge, and skill development, particularly regarding qualitative research methods: “It was a really great opportunity to learn about qualitative research and the methods that are used” (YAP member). Partnering with the YAP in manuscript preparation and the writing process invited critical discussions about authorship, credit, and power-sharing in research; topics that are often underexplored but central to authentic engagement. Further, involving the YAP in the early stages of manuscript development helped enforce their ownership over the final products and nurture trust and mutual respect between the patient partners and research team. This ultimately fostered a positive and sustainable partnership that has resulted in ongoing research collaborations and opportunities for mentorship and capacity-building.

### 3.7 Patient partner engagement in post-trial implementation and dissemination

As the team begins planning for the post-trial implementation of the intervention at additional sites throughout Canada, there is a plan to engage AYAs to provide consultation guidance. In refining the e-learning modules and the delivery of the program (e.g. referral process), Crohn's and Colitis Canada, the implementing organization, intends to create an engagement strategy to seek perspectives of a cross-section of AYAs. This will include the YAP members from this project and the recruitment of additional AYAs at various levels of engagement. Consultation feedback from YAP members that could not be integrated mid-trial will be considered in the refinement process before the intervention's scale-up. This could include incorporating badges for module completion and gamification tools to improve user experience and encourage module completion. While these additions were suggested during study execution, it was not feasible to implement changes mid-trial. This emphasizes the importance of engaging AYA patient partners early on and throughout all study phases to ensure insights are integrated promptly.

## 4 Patient engagement reflections and lessons learned

A series of learnings were obtained through an active partnership between academic researchers and the YAP, who formed the study team for the IBD-TrAYN Trial. Reflections regarding the YAP’s structure, co-designed engagement framework, motivations for involvement, and knowledge acquisition are described below. This is followed by a summary of the lessons we learned about patient engagement throughout the RCT.

### 4.1 Community-based structure of the youth advisory panel

Many YAP members stated that the inherent community-based structure of the YAP helped sustain engagement and promote a collaborative environment that encouraged all voices. More specifically, this forum offered YAP members the motivation, community support, and encouragement to engage in thought-provoking discussions where ideas could be shared, challenged, and formed in a safe, welcoming, and inclusive space. Observing how virtual group interactions stimulate creativity and invite members to develop innovative solutions and insights that otherwise may not have emerged in a more individualized and fragmented effort emphasized the importance of fostering an environment conducive to open communication, collaboration, and learning. The frequent and integrative use of icebreaker strategies in scheduled consultations, especially in the early stages of our collaborative relationship, was essential in addressing some of the power dynamics inherent in research team engagement with AYA patient partners.

### 4.2 Co-creating an engagement framework with AYA patient partners

The hierarchical nature of research collaboratives, where patient partners often perceive their role as peripheral or of low priority, can make it challenging to establish balanced power partnerships. AYA patient partners may feel restricted in their ability to contribute meaningfully. Partnerships may represent tokenism, decoration, or manipulation.^9^ Establishing the YAP charter for our RCT enabled the organic and unrestricted integration of patient partners’ roles across different study phases, inviting YAP members to participate in higher-level decision-making from research conceptualization throughout. AYA patient partners' contributions to the YAP charter helped establish an individualized engagement framework for the RCT by clarifying roles, responsibilities, expectations, and aspirations for the research team and partners. A YAP member even stated that “having the opportunity to define [their] position in the council and the wider research team was an empowering experience that helped foster a sense of purpose, ownership, and commitment to the study”.

### 4.3 Mapping AYA patient partners’ intrinsic & extrinsic motivations for involvement

**Fig. 2:**
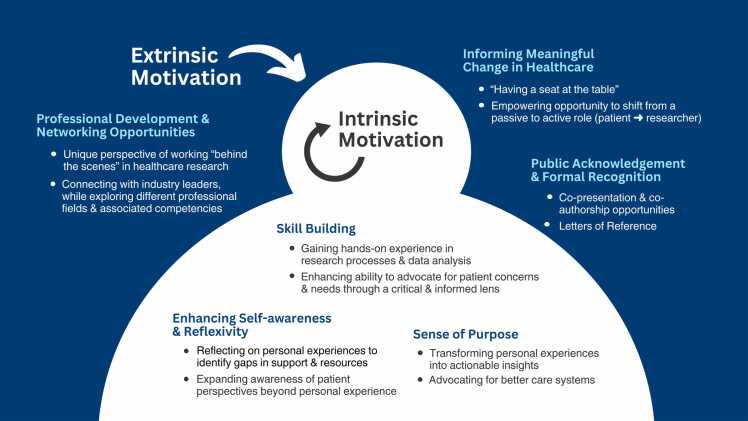
Patient Partners’ Motivations for Involvement in Research.

Mapping AYA patient partners’ motivations for engagement showed that their motivations could be broadly divided into two general categories: intrinsic and extrinsic (see Fig. 2). Three key intrinsic motivations associated with internal satisfaction and personal fulfillment constructed through our analysis included: i) the prospect of skill building, ii) discovering and pursuing a sense of purpose, and iii) the potential for enhancing self-awareness and reflexivity. YAP members curious or passionate about healthcare research viewed the YAP as an opportunity to learn about different research methodologies while enhancing their communication, leadership, and advocacy skills. Members who identified as patient/health advocates viewed the YAP as an opportunity to honour their commitment to ensuring that research about healthcare transitions or IBD remains grounded in the realities of those it impacts. Engaging in reflexive discussions about healthcare transition in IBD allowed YAP members to consider how their experiences compared to AYAs from other backgrounds, and to identify knowledge gaps that could inform future research studies.

The three key extrinsic motivations aligning with external rewards and incentives included: i) professional development and networking opportunities, ii) informing meaningful, lasting change in healthcare, and iii) public acknowledgement and formal recognition. Members aspiring to pursue careers in academia or healthcare mentioned that gaining hands-on experience in research processes and data analysis techniques was essential in gaining a “behind-the-scenes" look at different professional fields and associated competencies. One patient partner appreciated moving beyond the passive recipient of care to an active partner in the research process and a steward in their health journey. Lastly, patient partners reported that receiving public acknowledgement and formal recognition through co-presentation, co-authorship opportunities and letters of reference were great incentives to engage meaningfully and consistently with a multidisciplinary team across different study phases.

### 4.4 Supporting AYA patient partners in building foundational knowledge to enhance engagement

The delivery of high-level introductory presentations by the research team on fundamental concepts such as the study’s design, background, primary objectives, and future directions was essential in setting a solid foundation and providing AYA patient partners with clear contexts to meaningfully apply their lived experience within the study’s established framework. YAP members admitted that this “big picture” understanding of the project better positioned them to think more critically and reflect on where their perspectives would add the most value, adding nuance and depth to the research process. Further, the acquisition of knowledge and the opportunity to reflect on one’s healthcare transition journey were noted as impactful on patient partners’ growth and development, as described in Textbox 1.

Textbox 1 **YAP member reflections on the impact of engagement.**

“Reflecting on my own transition experience deepened my understanding of the challenges I faced during my transition to adult care, leading to greater self-awareness and clarity about my journey as an IBD patient. This experience also made me more aware of existing gaps in support and resources, highlighting areas of improvement needed to facilitate smoother healthcare transitions for others. Engaging with other patient partners and narratives collated from the semi-structured interviews helped me develop a stronger sense of empathy, allowing me to connect more deeply with their experiences and develop a more comprehensive understanding of patient perspectives from a broader perspective. Actively contributing to different stages of the research process and study, both as a patient partner and an aspiring physician helped reinforce my commitment to advocating for better care systems, while enhancing my ability to effectively communicate patient concerns and needs through a critical lens. In other words, it gave me a sense of purpose knowing that my insights could help shape a more supportive and patient-centered transition for other patients with IBD." (YAP member 1)

“[Regarding] the aspect we're contributing to larger patient care, I think that's been a really important aspect of it for me, especially considering as a patient myself, usually you're the topic of discussion at these tables. So, the fact that now I have a seat at the table to be involved equally in contributing to this research project, I think that was a really neat opportunity. It's kind of like usually you're the person sitting in the crowd at a play, but we got to go behind the scenes of the play and figure out how everything works. And then that's what's being presented on stage. So, I think that was a really big aspect for me - just that ability to go from patient to researcher and the continuum there and understand that as much your experiences are contributing to your seat at that table was just a really, really meaningful experience for myself.” (YAP member 2)

### 4.5 Lessons learned about patient engagement

**Fig. 3:**
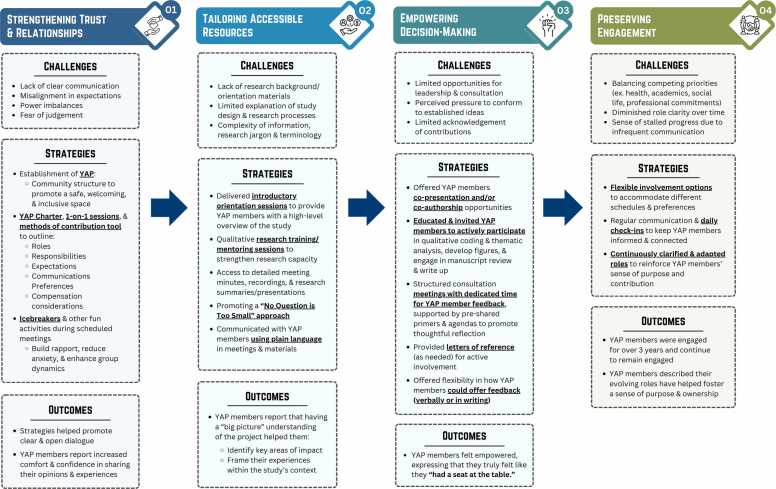
S.T.E.P. framework outlining learnings about patient engagement.

To encapsulate our lessons learned about patient engagement across different stages of the RCT, we conceptualized the S.T.E.P. framework, representing the vital importance of: i) strengthening trust and relationships, ii) tailoring accessible resources, iii) empowering decision-making, and iv) preserving meaningful engagement (see Fig. 3). This figure outlines challenges commonly faced by patient partners and research teams in each domain, mitigating strategies adopted in our partnership to address barriers, and their subsequent impact on patient engagement and outcomes.

### 4.6 Future directions

The involvement of an AYA with lived experience, first as an AYA patient partner involved in priority setting and later as a staff member on the project (managerial role), ensured AYA voices were embedded throughout the trial. Exploring the possibilities, multidirectional benefits, and implications of patient partners in paid positions on research teams is an important future direction in the patient engagement landscape. Subsequent research could aim to incorporate evaluation measures into engagement structures from the outset of RCTs, ideally co-developed with patient partners, to adequately capture and report on what is meaningful to them. Bringing the voices of AYAs with IBD from equity-deserving groups and from remote and rural communities should be a priority to ensure their perspectives inform research processes and outputs. Finally, establishing a national network of AYA patient partners in Canada to recognize the crucial role of people with lived experience driving progress and inspiring change in IBD research could be considered. Two AYA patient partners on our team (PM, CT) are working towards this goal, intending to create engagement mechanisms to ensure that research is responsive to the needs of those with IBD.

## 5 Conclusion

AYA patient partners’ contributions to this RCT were impactful, from study conceptualization to dissemination. Their involvement ensured the intervention's relevance, usability, and youth-friendliness, informed data analysis, and enhanced the knowledge translation outputs through co-creation. In addition to influencing the study materials and outputs, the engagement process impacted the study team, including the AYA patient partners, through meaningful collaboration and reciprocal learning opportunities.

## Ethics Statement

Ethical approval for the IBD-TrAYN Trial was granted by the Research Ethics Boards of The Hospital for Sick Children (REB #1000078476), Children's Hospital of Eastern Ontario (21/143X), and BC Children's Hospital (H21–03541).

## Funding Sources

This study was supported by a grant from the 10.13039/100007028Leona M. and Harry B. Helmsley Charitable Trust (Grant #2103-05041) and Crohn’s and Colitis Canada. This study was also supported by The Canadian Children IBD Network (with funding provided by the CH.I.L.D. Foundation). The funding sources did not support the study design, collection, data analysis and interpretation, manuscript preparation, or the decision to submit the article for publication. Eric I. Benchimol holds the Northbridge Financial Corporation Chair in Inflammatory Bowel Disease, a joint Hospital-University Chair between the 10.13039/501100003579University of Toronto, The Hospital for Sick Children, and the SickKids Foundation.

## Author Credentials

Brooke Allemang, PhD, MSW, RSW, Ashleigh Miatello, MA, Pranshu Maini, Joshua Eszczuk, Claudia Tersigni, MSc, Samantha Micsinszki, PhD, Sneha Dave, Natasha Bollegala, MD, MSc, FRCPC, Nancy Fu, MD, MHSc, Kate Lee, PhD, MBA, Samantha J. Anthony, PhD, MSW, RSW, Melanie Barwick, PhD, Eric I. Benchimol, MD, PhD

## CRediT authorship contribution statement

**Joshua Eszczuk:** Writing – review & editing, Formal analysis. **Pranshu Maini:** Writing – original draft, Formal analysis. **Eric I. Benchimol:** Writing – review & editing, Supervision, Resources, Funding acquisition. **Ashleigh Miatello:** Writing – review & editing, Methodology, Investigation, Formal analysis, Conceptualization. **Melanie Barwick:** Writing – review & editing, Supervision. **Allemang Brooke A.:** Writing – original draft, Methodology, Investigation, Formal analysis, Conceptualization. **Samantha J. Anthony:** Writing – review & editing, Supervision, Conceptualization. **Kate Lee:** Writing – review & editing. **Nancy Fu:** Writing – review & editing. **Natasha Bollegala:** Writing – review & editing. **Sneha Dave:** Writing – review & editing. **Samantha Micsinszki:** Writing – review & editing. **Claudia Tersigni:** Writing – review & editing.

## Declaration of Competing Interest

The authors declare the following financial interests/personal relationships which may be considered as potential competing interests: Eric I. Benchimol reports financial support was provided by Leona M and Harry B Helmsley Charitable Trust, Crohn’s and Colitis Canada, The Canadian Children IBD Network. Eric I. Benchimol reports a relationship with McKesson Canada that includes: consulting or advisory. Eric I. Benchimol reports a relationship with Dairy Farmers of Ontario that includes: consulting or advisory. Eric I. Benchimol reports a relationship with Canadian Agency for Drugs and Technologies in Health that includes: consulting or advisory. If there are other authors, they declare that they have no known competing financial interests or personal relationships that could have appeared to influence the work reported in this paper.

## Data Availability

Data will be made available on request.

## Footnotes

Appendix A Supplementary data associated with this article can be found in the online version at doi:10.1016/j.hctj.2025.100111.
