# Supplementary material for: Reflections on youth partnership in a randomized controlled trial of an intervention to optimize transition from pediatric to adult care in inflammatory bowel disease
Source: Health Care Transit. 2025 Jul 5;3:100111. doi: 10.1016/j.hctj.2025.100111 (PMC12272582; doi:10.1016/j.hctj.2025.100111)
Supplement: Supplementary file 1 — Supplementary material [file mmc1.docx]

Supplementary Table 1: International Association for Public Participation (IAP2) Spectrum of Public Participation and Corresponding Extent of Patient Involvement

| **Type of Engagement (IAP2 Levels)** | **Extent of Patient Involvement** |
| --- | --- |
| Inform | Very minimal – patients are only receiving information |
| Consult | Low – patients provide input but don’t influence decisions |
| Involve | Moderate – patient input is considered more actively |
| Collaborate | High – patients are partners in decision-making |
| Empower | Very high – patients lead decisions or have final authority |
